# Global Landscapes of the *Na^+^/H^+^ Antiporter (NHX)* Family Members Uncover their Potential Roles in Regulating the Rapeseed Resistance to Salt Stress

**DOI:** 10.3390/ijms21103429

**Published:** 2020-05-12

**Authors:** Jia-qian Cui, Ying-peng Hua, Ting Zhou, Ying Liu, Jin-yong Huang, Cai-peng Yue

**Affiliations:** 1 School of Life Sciences, Zhengzhou University, Zhengzhou 450001, China; hyp19890413@163.com; 2 School of Agricultural Sciences, Zhengzhou University, Zhengzhou 450001, China; yingpenghua@zzu.edu.cn (Y.-p.H.); zhoutt@zzu.edu.cn (T.Z.); lyzzuniversity@163.com (Y.L.); jinyhuang@zzu.edu.cn (J.-y.H.)

**Keywords:** Allotetraploid, *Na^+^/H^+^ antiporter* (*NHX*), *Brassica napus*, genome-wide identification, salt stress

## Abstract

Soil salinity is a main abiotic stress in agriculture worldwide. The *Na^+^/H^+^* antiporters (*NHXs*) play pivotal roles in intracellular Na^+^ excretion and vacuolar Na^+^ compartmentalization, which are important for plant salt stress resistance (SSR). However, few systematic analyses of *NHXs* has been reported in allotetraploid rapeseed so far. Here, a total of 18 full-length *NHX* homologs, representing seven subgroups (*NHX1-NHX8* without *NHX5*), were identified in the rapeseed genome (A_n_A_n_C_n_C_n_). Number variations of *BnaNHXs* might indicate their significantly differential roles in the regulation of rapeseed SSR. *BnaNHXs* were phylogenetically divided into three evolutionary clades, and the members in the same subgroups had similar physiochemical characteristics, gene/protein structures, and conserved Na^+^ transport motifs. Darwin´s evolutionary pressure analysis suggested that *BnaNHXs* suffered from strong purifying selection. The cis-element analysis revealed the differential transcriptional regulation of *NHXs* between the model Arabidopsis and *B. napus*. Differential expression of *BnaNHX*s under salt stress, different nitrogen forms (ammonium and nitrate), and low phosphate indicated their potential involvement in the regulation of rapeseed SSR. Global landscapes of *BnaNHXs* will give an integrated understanding of their family evolution and molecular features, which will provide elite gene resources for the genetic improvement of plant SSR through regulating the *NHX*-mediated Na^+^ transport.

## 1. Introduction

Plants are usually exposed to various environmental stresses, among which salinity is one of the major limiting factors for plant growth and development, and crop productivity [1,2,3]. Approximately 50% of the irrigated land worldwide suffers from salinity damage, which causes osmotic stress and ion disorder in plants [4]. In detail, salt stresses inhibit seed germination, root growth, photosynthesis and crop fructification [5].

Plants have developed multiple strategies to cope with salt stresses, including the regulation of growth and development, ion homeostasis, detoxification and osmotic adjustment [5]. Among them, the maintenance of ion homeostasis plays an essential role in the plant salt stress resistance (SSR). Under salt stresses, the decrease in sodium ion (Na^+^) concentrations and the increase in potassium ion (K^+^) concentrations, leading to a high K^+^/Na^+^ ratio, are very crucial for plant SSR. In this process, the Na^+^/H^+^ antiporters (NHXs) are very important players. Most NHXs, localized on the plasma membrane and tonoplast, are key to the maintenance of low intracellular Na^+^ concentrations by discharging Na^+^ into the extracellular parts and vacuole [6]. NHXs are driven by proton (H^+^) electrochemical gradients that are generated by two different types of H^+^ pumps: H^+^-ATPase and H^+^-PPase [7,8]. Arabidopsis *NHX*s can be divided into three main groups: (i) *AtNHX1-4* localized on the tonoplast, (ii) *AtNHX5-6* localized on the endosomal compartment, and (iii) *AtSOS1* (*Salt Overly Sensitive 1*)*/NHX7* and *AtNHX8* localized on the plasma membrane [9,10,11]. *NHXs* are involved in cell expansion [12], pH regulation [13], SSR [14], K^+^ homeostasis [15,16], Na^+^ long-distance transport [17], and other physiological processes [18,19,20,21]. More and more studies are aimed at improving plant SSR through molecular modulation of *NHXs*. Enhanced expression of Arabidopsis *NHX1* in rapeseed and tomato improved the plant SSR [22,23]. The silencing of *GhNHX1* resulted in the enhanced sensitivity of cotton seedlings to high salt concentrations, which suggested that *GhNHX1* positively regulated the cotton resistance to salt stress [24]. Ectopic expression of a wheat antiporter gene (*TaNHX2*) improved the SSR and growth performance in transgenic sunflower plants [25]. Ectopic expression of Arabidopsis *NHX5* enhanced the soybean SSR [26].

Oilseed rape (*Brassica napus* L.) is widely cultivated and harvested for the production of vegetable oil, livestock protein powder, and biodiesel [27]. The allotetraploid rapeseed (A_n_A_n_C_n_C_n_, 2n=4x=38) originates from spontaneous hybridization of its diploid ancestors *Brassica rapa* (A_r_A_r_, 2n=2x=20) and *Brassica oleracea* (C_o_C_o_, 2n=2x=18) [28,29,30]. The seed yield and quality of rapeseed is also inhibited by soil salinity [31]. The whole-genome study of *NHXs* provides important information for the elucidation of molecular mechanisms underlying Na^+^ homeostasis and plant SSR. However, the *NHX* family has not been systematically studied in allotetraploid *B. napus*. Moreover, the core members have also been unknown among the numerous *BnaNHXs*. Therefore, in this study, we aimed to: (i) identify the genome-wide *NHXs* and core members in *B. napus*; (ii) determine the gene and protein features of *BnaNHXs*; and (iii) analyze the transcriptional responses of *BnaNHXs* to salt stress and other nutrient stresses, including ammonium toxicity and low phosphate. The genome-wide identification and molecular characterization of *NHXs* indicated their evolutionary conservation and functional divergence between allotetraploid rapeseed and the model Arabidopsis. Global landscapes of *BnaNHXs* will give an integrated understanding of their roles in the regulation of plant SSR, which will provide elite gene resources for the genetic improvement of plant SSR through regulating the *NHX*-mediated Na^+^ transport.

## 2. Results

### 2.1. Genome-Wide Identification of NHXs in Plant Species

**Figure 1:**
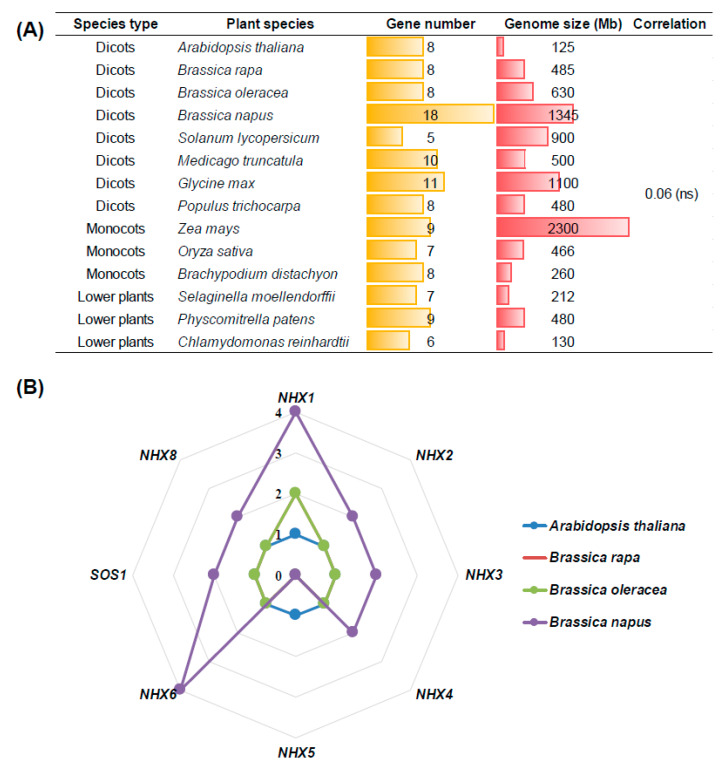
Copy number of the *Na+/H+ antiporter* (*NHX*) family members in plant species. (**A**) Comparison of the total gene number of the *NHX* family in plant species. The correlation refers to the relationship between the *NHX* family gene numbers and plant genome sizes. ns: not significant. (**B**) Each *NHX* subgroup member number in Arabidopsis and three *Brassica* crops (*Brassica rapa*, *Brassica oleracea*, and *Brassica napus*).

To identify the *NHX* family members in diverse plant species, we used the amino acid sequences of Arabidopsis NHXs as queries to perform BLASTp search against the genome databases of *B. rapa* (‘Chiifu-401’), *B. oleracea* (‘TO1000’), *B. napus* (‘Darmor-*bzh*’), and other plant species. We found great differences in the homolog number of *NHXs* in dicots, monocots, and lower plant species (Table 1, Figure 1A). The plant genome sizes varied from 125 Mb (*Arabidopssis thaliana*) to 2300 Mb (*Zea mays*), and the *NHX* number ranged from 5 (*Solanum lycopersicum*) to 18 (*B. napus*). On the whole, the *NHX* homolog numbers appeared not to be closely correlated with the plant genome sizes (correlation coefficient = 0.06 > *p* = 0.05). In the model *A. thaliana*, *NHXs* had eight members (*NHX1-NHX8*), namely, each *NHX* member only had a single copy. We identified a total of 8, 8, and 18 *NHX* homologs in the diploid *B. rapa*, *B. oleracea*, and the allotetraploid *B. napus*, respectively. The homolog number of *NHXs* in *B. napus* was similar to the sum of *NHXs* in *B. rapa* and *B. oleracea* (Table 1). Therefore, it might be concluded that most *NHXs* were kept during the spontaneous hybridization between *B. rapa* and *B. oleracea* for the formation of allotetraploid *B. napus*. In detail, both *BnaNHX1s* and *BnaNHX6s* had four homologs, and *BnaNHX2s*, *BnaNHX3s*, *BnaNHX4s*, *BnaSOS1s/BnaNHX7s*, and *BnaNHX8s* had two homologs in *B. napus* (Figure 1B). However, we found that *NHX5* was lost in *B. rapa*, *B. oleracea* and *B. napus* (Figure 1B). The variations in the *BnaNHX* number might indicate their significant differential roles in the resistance of *B. napus* to salt stresses.

### 2.2. Genomic Distribution and Expansion Patterns of BnaNHXs

**Figure 2:**
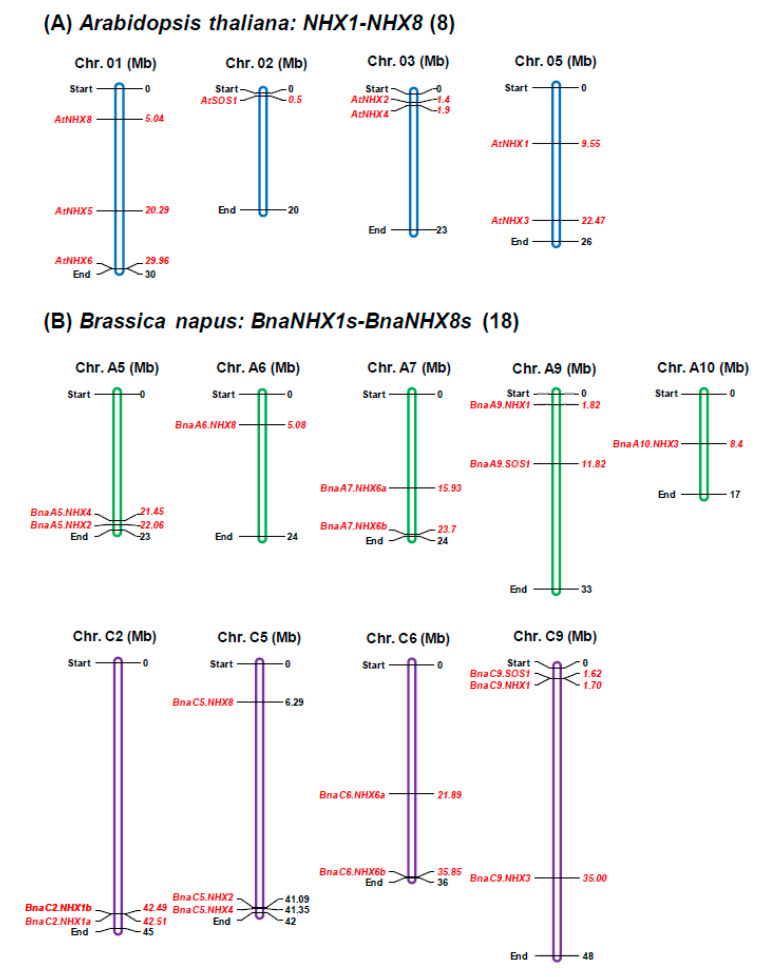
Physical mapping of *the Na+/H+ antiporter* (*NHX*) family members in *Arabidopsis thaliana* and *Brassica napus*. (**A**–**B**) Genomic distribution of the Arabidopsis *NHXs* (**A**) and their homologs in *B. napus* (**B**).

Through physical position identification of eight *NHXs* in Arabidopsis, we found that four of them were distributed on Chr. 01 (*AtNHX5, AtNHX6*, and *AtNHX8*), Chr. 02 (*AtSOS1*), Chr. 03 (*AtNHX2* and *AtNHX4*), and Chr. 05 (*AtNHX1* and *AtNHX3*), respectively (Table 2, Figure 2A). To further understand the genomic distribution and expansion patterns of *BnaNHXs*, we retrieved the DNA sequences of *BnaNHXs* from the *Brassica* and CNS-Genoscope databases. The *BnaNHXs* were physically mapped onto nine chromosomes (A subgenome: A5, A6, A7, A9 and A10; C subgenome: C2, C5, C6 and C9) of *B. napus* (Table 2, Figure 2B). The 18 *BnaNHXs* were unevenly distributed in different chromosomes (Table 2, Figure 2). The chromosomes C9 and C5 had more *BnaNHXs*, including *BnaC9.NHX1* (BnaC09g02990D), *BnaC9.NHX3* (BnaC09g31970D), and *BnaC9.SOS1* (BnaC09g52290D) on C9, and *BnaC5.NHX2* (BnaC05g46790D), *BnaC5.NHX4* (BnaC05g45460D), and *BnaC5.NHX8* (BnaC05g10850D) on C5, than other chromosomes did (Table 2, Figure 2B).

Gene family expansion occurs mainly via four pathways: tandem duplication, segmental duplication, whole-genome duplication/polyploidization and replicative transposition [32]. The *B. napus* progenitor diploid (*B. rapa* and *B. oleracea*) genomes are ancient polyploids, and large-scale chromosomal rearrangements have occurred since their evolution from the model Arabidopsis, a lower chromosome number progenitor [33]. Comparative genomics reveals that the Arabidopsis genome can be divided into 24 ancestral crucifer blocks, which are labeled A-X [34]. Table 2 shows that *AtNHXs* and their corresponding *BnaNHX* homologs were located on the same chromosomal blocks. In detail, the *NHXs* were located on the Q (*NHX1s*), F (*NHX2s*), W (*NHX3s*), F (*NHX4s*), C (*NHX5s*), E (*NHX6s*), K (*NHX7s/SOS1s*), and A (*NHX8s*) chromosomal blocks, respectively (Table 2). From the genomic distribution of *BnaNHXs*, we propose that the family expansion of *BnaNHXs* was mainly attributed to whole-genome duplication and segmental duplication, except *BnaC2.NHX1a* and *BnaC2.NHX1b*, which were derived from tandem duplication (Figure 2B).

### 2.3. Phylogeny Analysis of BnaNHXs

**Figure 3:**
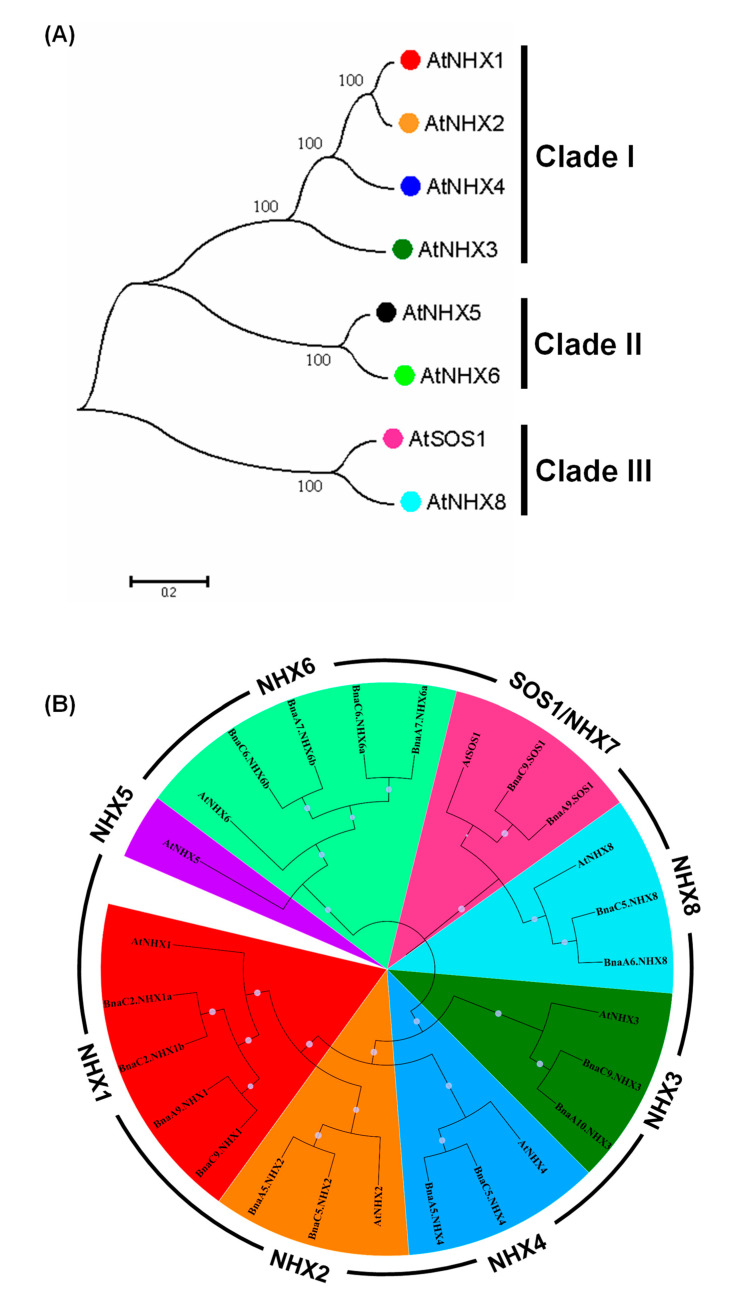
Phylogeny analysis of the Na+/H+ antiporters (NHXs) in *Arabidopsis thaliana* and *Brassica napus*. (**A*–*B**) Phylogeny analysis of AtNHXs (**A**) and the NHX homologs in *B. napus* (**B**). The NHX protein sequences were multi-aligned using the ClustalW program, and then an unrooted phylogenetic tree was constructed using the software MEGA 6.06 with the neighbor-joining method. The percentages of replicate trees, in which the associated taxa clustered together in the bootstrap test (1000 replicates), are shown next to the branches. The tree is drawn to scale, with branch lengths in the same units as those of the evolutionary distances used to infer the phylogenetic tree. The evolutionary distances were computed with the Poisson correction method and are in the units of the number of amino acid substitutions per site.

To elucidate the molecular evolution and phylogenetic relationships among the NHX proteins in Cruciferae species, we constructed unrooted phylogenetic trees involving AtNHXs and their homologs in *Brassica* species (Figure 3). In Arabidopsis, the NHX family members were mainly classified into three clades: Clade I (AtNHX1, AtNHX2, AtNHX3 and AtNHX4), Clade II (AtNHX5 and AtNHX6), and Clade III (AtSOS1/AtNHX7 and AtNHX8) (Figure 3A). Further, we performed a phylogeny analysis of 8 NHXs in *A. thaliana* and 18 NHXs in *B. napus*. The phylogenetic tree could also be three larger clades, which could be further classified into eight smaller categories, and each BnaNHX member was closely clustered with the corresponding homologs in *A. thaliana* (Figure 3B). The result indicated that the NHXs diverged before the *Brassica* speciation. Most of the NHXs within each subfamily had very short branch lengths (Figure 3B), which suggested the occurrence of recent genetic divergence.

### 2.4. Molecular Characterization of BnaNHXs

To understand the molecular characteristics of BnaNHXs, we calculated the physicochemical parameters of each BnaNHX using ExPASy. The results showed that most proteins in the same NHX subfamily had similar parameters (Table 2). In total, the CDS lengths of *BnaNHXs* varied from 1590 bp (*BnaA10.NHX3*) to 3420 bp (*BnaA9.SOS1* and *BnaC9.SOS1*), and the corresponding numbers of deduced amino acids varied from 529 to 1139 (Table 2). Most of the computed molecular weights of BnaNHXs ranged from 58.7 kD (BnaC9.NHX3) to 125.8 kD (BnaC9.SOS1 and BnaA9.SOS1) (Table 3). The theoretical isoelectric points (pIs) of BnaNHXs varied from 5.62 (BnaA7.NHX6a) to 7.76 (BnaA9.NHX1), with most >7.0 except that of BnaNHX6s (5.62–6.91) (Table 3). The grand average of hydropathy (GRAVY) value is defined as the sum of hydropathy values of the amino acids divided by the protein length. The results showed that BnaNHXs had GRAVY values ranging from 0.109 (BnaC9.SOS1) to 0.559 (BnaC9.NHX3) (Table 3), therefore all of which were assumed to be hydrophobic. Most instability indices of BnaNHXs were <40.0 (Table 3), and it indicated that most BnaNHXs showed strong protein stability, except those of BnaA7.NHX6a (41.65) and BnaC6.NHX6a (42.95), which were > 40.0. The online WoLF PSORT was used to predict the subcellular localization of 8 AtNHXs and 18 BnaNHXs. In general, the subcellular localizations of BnaNHXs were consistent with those of the corresponding Arabidopsis NHX homologs (Table 3, Figure S1). In detail, BnaNHX1s-4s were localized on the tonoplast, and BnaNHX5s and BnaNHX6s were localized on the endosome, while BnaSOS1s/BnaNHX7s and BnaNHX8s were localized on the plasma membrane. Different subcellular localizations of BnaNHXs indicated their distinct roles in the regulation of rapeseed SSR. To further identify the transmembrane topology of NHXs, we used the TMHMM tool to characterize their transmembrane structures, and found that AtNHXs and BnaNHXs had 8 to 12 membrane-spanning regions (Table 3; Figure S2). In detail, both AtNHX1 and BnaNHX1s had 12 transmembrane regions, and both AtNHX3 and BnaNHX3s had 11 transmembrane regions, whereas the other six subgroup members had different membrane-spanning regions between Arabidopsis and *B. napus* (Table 3). Prediction of phosphorylation sites in BnaNHXs showed that tyrosine is the most common site for phosphorylation (Figure S3). Similar to AtNHXs without signal peptides, BnaNHXs were not identified to have any signal peptides, either (Figure S4). The RPSP v. 2009 program predicted that the recombinant BnaNHX proteins would be insoluble when these proteins are overexpressed in *E. coli*.

### 2.5. Identification of Evolutionary Selection Pressure on BnaNHXs

To assess the selection pressure on *BnaNHXs* during the evolutionary process, we used the orthologous *NHX* pairs between *B. napus* and *A. thaliana* to calculate the values of Ka, Ks and Ka/Ks (Table 2). The Ka values of *BnaNHXs* ranged from 0.0338 (*BnaC2.NHX1a*) to 0.0909 (*BnaC9.SOS1*), with an average of 0.054, and the Ks values of *BnaNHXs* ranged from 0.2542 (*BnaA6.NHX8*) to 0.4996 (*BnaC9.NHX1*), with an average of 0.396. Further, we found that all the Ka/Ks values of *BnaNHXs* were < 1.0 (Table 2). Therefore, we proposed that *BnaNHXs* might have experienced very strong negative selection to preserve their functionality.

The Ks values of duplicated homologs among gene families are usually proposed to be molecular clocks, and they are expected to be similar over time. The segregation between the model Arabidopsis and its derived *Brassica* species occurred 12–20 million years ago (Mya) [35,36]. Our result showed that most *BnaNHXs* might diverge from *AtNHXs* approximately 12.0–18.0 Mya (Figure 3B), which implied that the *Brassica* plant speciation might be accompanied by the *NHX* divergence.

### 2.6. Conserved Domain, Gene Structure, Protein Interaction and Transcriptional Regulatory Analysis

**Figure 4:**
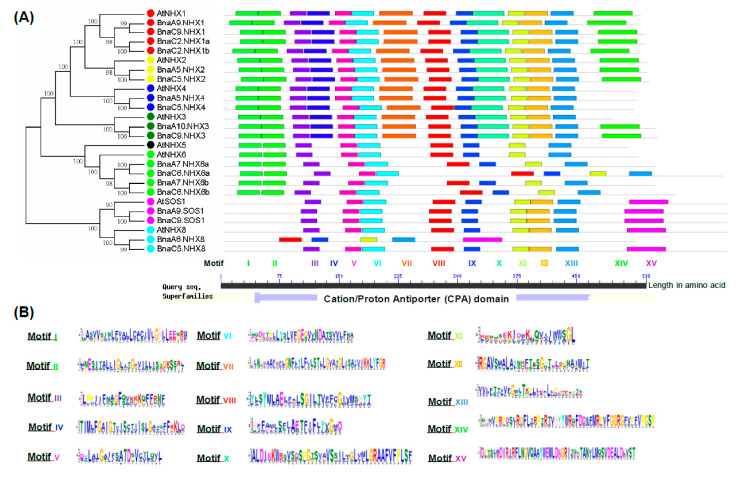
Identification and characterization of the conserved motifs in the Na+/H+ antiporters (NHXs) in *Arabidopsis thaliana* and *Brassica napus*. Molecular identification (**A**) and sequence characterization (**B**) of the conserved motifs in the NHXs. In Figure 4**A**, the boxes with different colors indicate different conserved motifs (motif I–XV), and grey lines represent the NHX regions without detected motifs. In Figure 4**B**, the larger the fonts, the more conserved the motifs.

The amino acid residues are thought to be functionally or structurally significant if they are evolutionarily conserved. The MEME results showed some conserved domains among the 8 AtNHXs and 18 BnaNHXs (Figure 4A). The predicted motifs of BnaNHXs ranged from 6 to 50 amino acids in length. Further, we identified that these conserved domains belonged to the Cation/Proton Antiporter (CPA) family (Figure 4A). Among the 15 conserved domains that we defined, we found that the amino acid sequences of the Motif III, V, VI, VIII, IX, XI and XIII had the highest identity among all the BnaNHXs (Figure 4B), and thus might be used as the indicators of the NHX family members. Noticeably, the amiloride-binding site (FFIYLLPPI), which is a typical feature of NHXs, was also found in the Motif II and III of BnaNHX1s-4s, whereas it was absent in BnaNHX6s-8s (Figure 4B).

**Figure 5:**
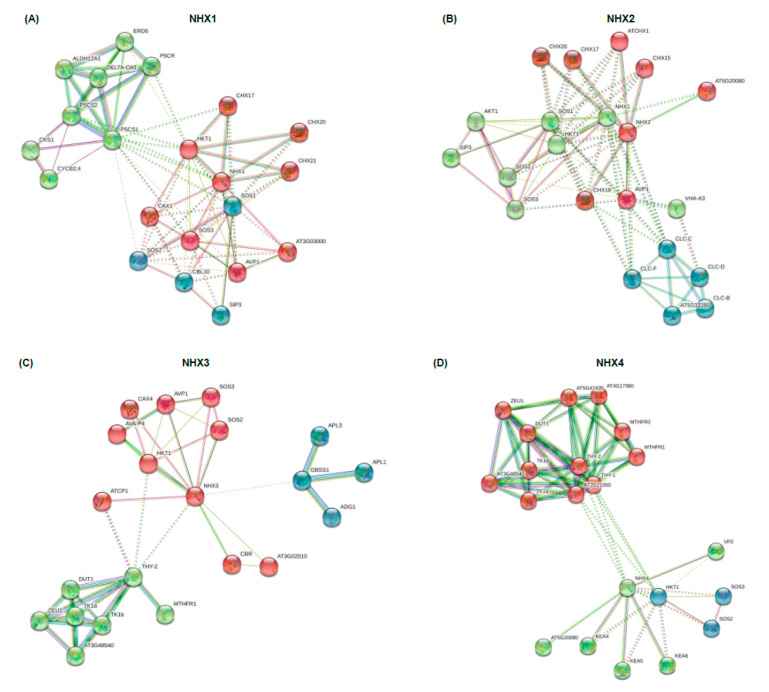

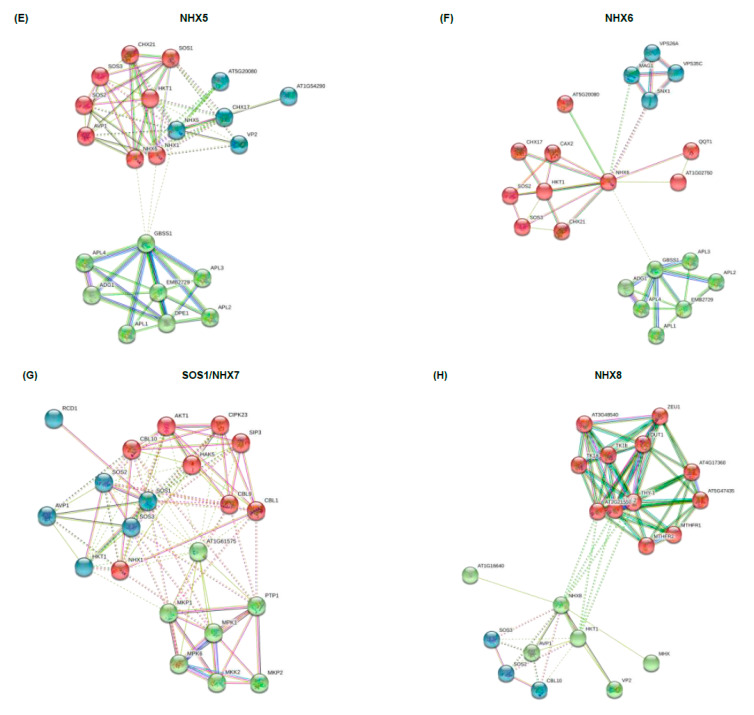
Protein–protein interaction networks involving the Na+/H+ antiporters (NHXs). The interaction networks of NHX1 (**A**), NHX2 (**B**), NHX3 (**C**), NHX4 (**D**), NHX5 (**E**), NHX6 (**F**), SOS1/NHX7 (**G**) and NHX8 (**H**) with other proteins were constructed by the STRING web-server. Network nodes represent proteins. Network is clustered to three clusters, which are represented with red, green and blue nodes, respectively. Colored nodes: query proteins and first shell of protein interactors; white nodes: second shell of protein interactors. Empty nodes: proteins of unknown 3D structure; filled nodes: some 3D structure is known or predicted. Edges represent protein–protein associations.

To further identify which proteins might interact with BnaNHXs, we constructed protein interaction networks using the STRING database, based on either known experimental or predicted interactions. A previous study has shown that the transport activity of NHX is regulated by SOS2 and SOS3 [37], and HKT1 can also transport Na^+^ [38]. Consistently, all the NHXs interacted with high-affinity K^+^ transporters (HKTs), calcineurin B-like protein 4 (CBL4/SOS3), and CBL-interacting protein kinase 24 (CIPK24/SOS2) (Figure 5). In addition, except NHX4 and NHX6, the other NHXs interacted with AVP1 (Figure 5), which serves a job-sharing role with V-ATPase in vacuolar acidification [39].

The secondary structures of NHXs were predicted by the Phyre2 software, and the alpha helix, beta strand, disordered and transmembrane helix were mainly analyzed (Supplementary Figure S5). The alpha helix percentage of the BnaNHX secondary structures ranged from 67% (BnaA9.SOS1) to 79% (BnaC6.NHX6b), with an average of 75%, which indicated that alpha helix was a major constituent of the BnaNHX secondary structure. However, beta strand was not found in most BnaNHXs, except BnaSOS1s. The disordered structure ratios of BnaNHXs ranged from 17% (BnaA6.NHX8) to 26% (BnaA9.SOS1), with an average of 19.39%. The transmembrane helix proportion of BnaNHXs ranged from 22% (BnaA9.SOS1/BnaC9.SOS1) to 55% (BnaA7.NHX6b), with an average of 43.22% (Figure S5).

The number and organization of exon–intron structures are indicative of evolutionary imprints within gene families. Therefore, it led us to identify the *BnaNHX* structures, through the comparison of the genomic DNA sequences, with their corresponding coding sequences. As shown in Table 2, in general, most *BnaNHXs* had similar gene structures to Arabidopsis *NHXs*, which suggested their conserved functionality between the ancestor Arabidopsis and the progenitor *B. napus*. However, we also observed some structure variations between the same *BnaNHX* subgroup. The number of exons and introns of *BnaNHX6s* and *BnaNHX8s* was different from those of *AtNHX6* and *AtNHX8*, respectively (Table 2, Figure S6). The exon number changes, potentially caused by alternative splicing, might contribute to the functional differentiation among different *BnaNHXs*.

**Figure 6:**
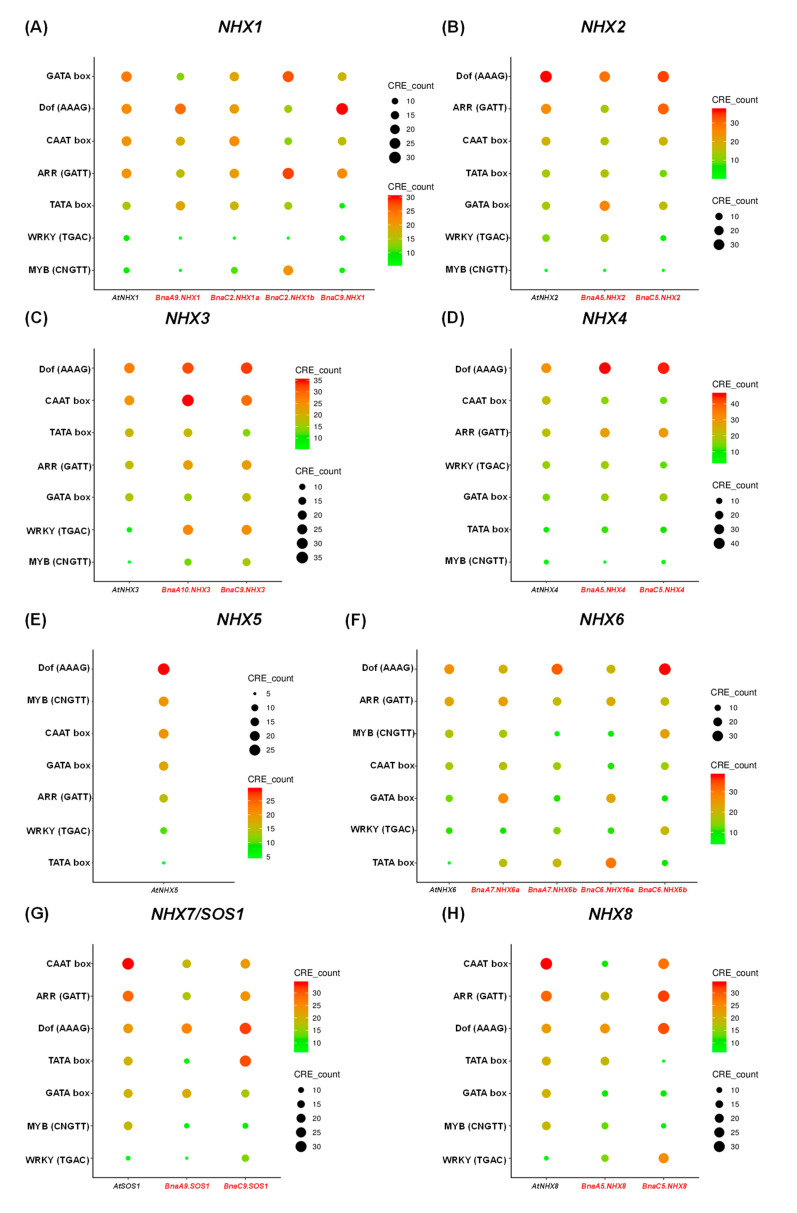
Enrichment analysis of the cis-acting regulatory elements (CREs) in the gene promoter regions of *Na+/H+ antiporters (NHXs)* in *Brassica napus*. Enrichment analysis of the CREs in the promoter regions of *NHX1s* (**A**), *NHX2s* (**B**), *NHX3s* (**C**), *NHX4s* (**D**), *AtNHX5* (**E**), *NHX6s* (**F**), *NHX7s/SOS1s* (**G**), and *NHX8s* (**H**). In the scatter plot, the larger the circle size, the more the corresponding CREs.

Transcription factors, binding to the cis-acting regulatory elements (CREs) in the promoter regions of their target genes, play important roles in the transcriptional regulation [40]. To identify the core transcription factors regulating *NHXs*, the 2.0-kb upstream region sequences of the *BnaNHX* start codons were used to explore the enriched CREs (Figure 6). The results showed that *BnaNHX*s had various types of CREs in their promoter regions, and suggested that complicated regulatory networks may be involved in the transcriptional regulation of *BnaNHXs*. Apart from the common CREs, such as the TATA box and the CAAT box, we also identified that the CAAT-box, DNA-binding with One Finger (Dof, AAAG), and Age-Related Resistance (ARR, GATT) elements were three highly enriched terms in the *BnaNHX* promoters (Figure 6). Compared with the CREs in the *BnaNHX* promoters, the most over-presented CREs were the DNA-binding with one finger (Dof), MYB-binding and MYC/bHLH-binding elements, although some common CREs, including Dof (AAAG), CAAT-box and ARR (GATT), were also identified in both Arabidopsis and *B. napus* (Figure 6). These results showed that there existed common and specific regulatory mechanisms in the *NHX*s of Arabidopsis and *B. napus*.

### 2.7. Transcriptional Analysis of BnaNHXs Under Diverse Nutrient Stresses

To identify the roles of *BnaNHXs* in regulating rapeseed against salt stress, we explored their transcriptional responses under these circumstances. First, we investigated the transcriptional patterns of *AtNHXs* in various tissues through the TAIR eFP Browser. The results showed that *AtNHX1*, *AtNHX2*, *AtNHX3* and *AtNHX4* were highly expressed in the vacuole (Figure S1), which indicated that they might play key roles in the transport of Na^+^ from the cytoplasm into the vacuole. In addition, both *AtNHX5* and *AtNHX6* had the highest expression levels in the endosome (Figure S1). *AtSOS1/AtNHX7* and *AtNHX8* had preferential expression in the plasma membrane (Figure S1). The differential expression characteristics implied the specific roles of *NHXs* in the regulation of plant SSR.

The results above-mentioned indicated that multicopies of each *NHX* homolog occurred in allotetraploid rapeseed (Figure 1, Table 1), and that transcriptional identification of the core *NHX* members is very important for a more in-depth understanding of *BnaNHXs*, which would contribute to the identification of elite gene resources for the genetic improvement of rapeseed SSR. 

**Figure 7:**
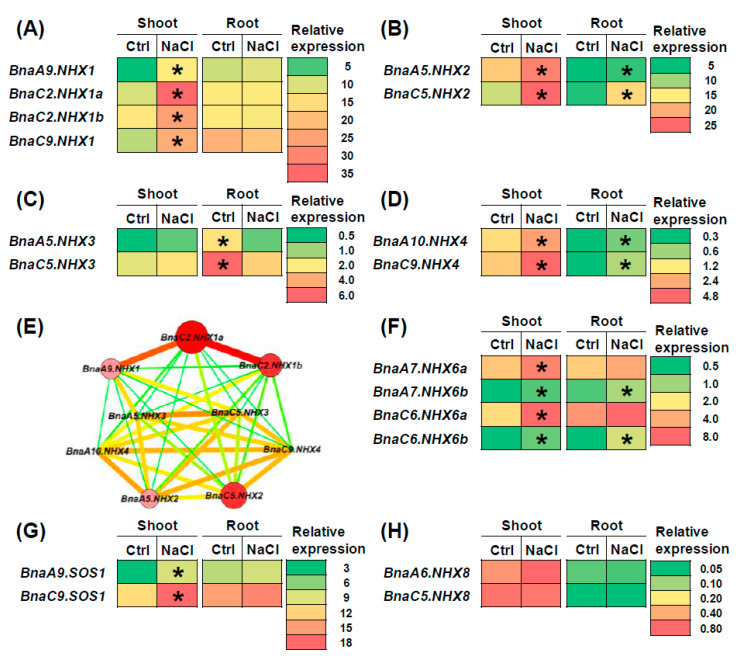
The qRT-PCR assisted transcriptional characterization of the *Na+/H+ antiporters* (*NHXs*) in *Brassica napus* under salt stress. (**A**–**D**) Differential expression profiling of: *BnaNHX1s* (**A**), *BnaNHX2s* (**B**), *BnaNHX3s* (**C**), and *BnaNHX4s* (**D**) between the control (Ctrl) and salt stress. (**E**) The gene co-expression network diagram involving *BnaNHX1*s, *BnaNHX2s*, *BnaNHX3s*, and *BnaNHX4s*. (**F**–**H**) Differential expression profiling of *BnaNHX6s* (**F**), *BnaSOS1s/BnaNHX7s* (**G**), and *BnaNHX8s* (**H**) under salt stress. For the transcriptional analysis, the 7 d-old uniform *B. napus* seedlings after seed germination were hydroponically cultivated in a NaCl-free solution for 10 d, and then were transferred to 200 mM NaCl for 12 h until sampling. The shoots and roots were individually sampled, and each sample includes three independent biological replicates. The significance level of *p* < 0.05 is used as the threshold to identify the differential *BnaNHX* expression under salt stress. The differentially expressed genes with higher expression are indicated with asterisks.

A total of 16 members of the 18 *BnaNHXs*, except 2 *BnaNHX8s* (*BnaA6.NHX8s* and *BnaC5.NHX8s*), were identified to be differentially expressed in rapeseed plants under salt stress (200 mM NaCl) (Figure 7). However, different *BnaNHX* members showed distinct transcriptional responses to salt stress. In detail, most (87.5%) of the differentially expressed genes (DEGs) were upregulated in the shoots or roots under salt stress (Figure 7), except that the two *BnaNHX3s* were downregulated in the roots (Figure 7H). Differential expression of *BnaNHX1s*, *BnaA7.NHX6a*, *BnaC6.NHX6a* and *BnaSOS1s* was only found in the shoots (Figure 7A,G). *BnaNHX2s*, *BnaA7.NHX6b*, *BnaC6.NHX6b* and *BnaNHX4s* were induced in both the shoots and roots (Figure 7B,D). To determine the core members functioning in the vacuolar sequestration of excessive Na^+^, we performed a co-expression network analysis of *BnaNHX1s-4s*. The result showed that *BnaC2.NHX1a* might play a major role in the vacuolar sequestration-mediated SSR of rapeseed plants (Figure 7E). *BnaC6.NHX6a* showed the highest expression levels among the four differentially expressed *BnaNHX6s*, and it was proposed to be the central gene in the endosome-mediated SSR (Figure 7E). In the plasma membrane-localized *SOS1/NHX7*- and *NHX8*-mediated Na^+^ efflux, *BnaC9.SOS1* was identified to be the core member, by virtue of its differential expression in response to salt stress and higher expression levels (Figure 7G,H).

**Figure 8:**
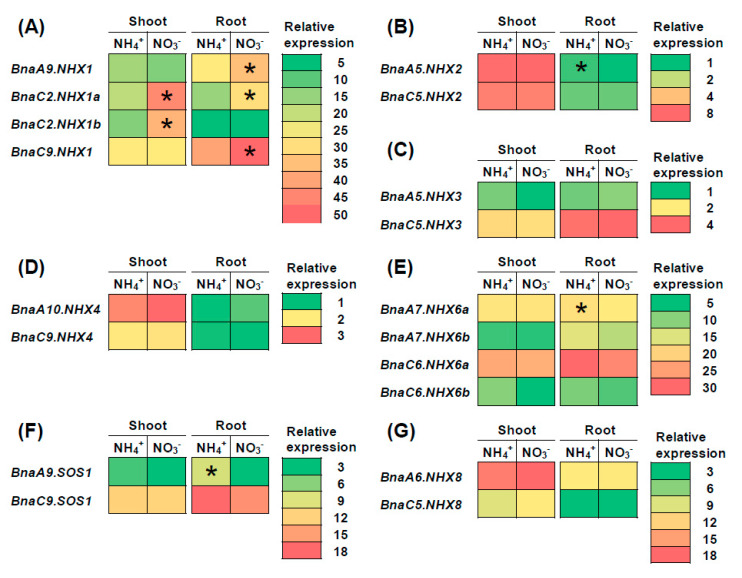
The qRT-PCR-assisted transcriptional characterization of the *Na+/H+ antiporters* (*NHXs*) in *Brassica napus* under different forms of nitrogen (N) conditions. Differential expression of *BnaNHX1s* (**A**), *BnaNHX2s* (**B**), *BnaNHX3s* (**C**), *BnaNHX4s* (**D**), *BnaNHX6s* (**E**), *BnaNHX7s* (**F**), and *BnaNHX8s* (**G**) under nitrate (NO_3_^-^) and ammonium (NH_4_^+^) conditions. For the transcriptional analysis, the rapeseed seedlings were first hydroponically cultivated under 6.0 mM nitrate (NO_3_^-^) for 10 d, and then were transferred to an N-free solution for 3 d. Subsequently, the above seedlings were sampled after treatment with 6.0 mM ammonium (NH_4_^+^) for 3 d. The shoots and roots were individually sampled, and each sample includes three independent biological replicates. The significance level of *p* < 0.05 is used as the threshold to identify the differential *BnaNHX* expression under NO_3_^-^ and NH_4_^+^ conditions. The differentially expressed genes with higher expression between different treatments are indicated with asterisks.

**Figure 9:**
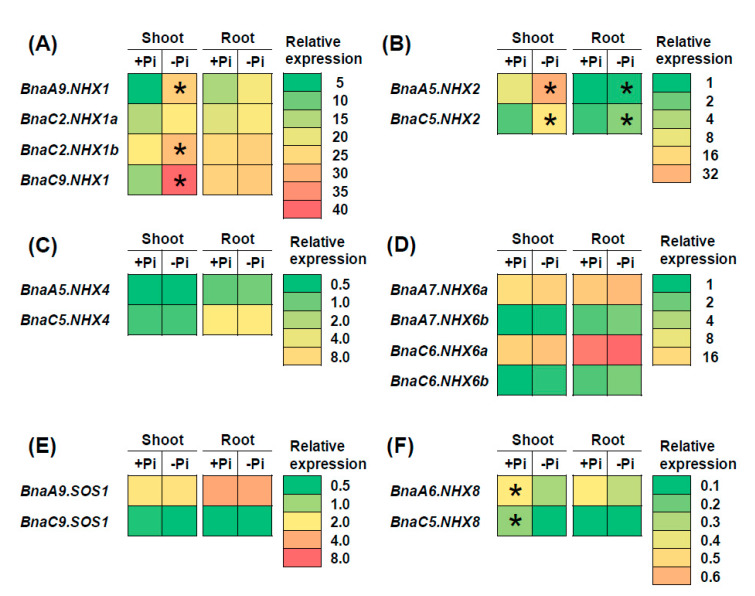
The qRT-PCR-assisted transcriptional characterization of the *Na+/H+ antiporters* (*NHXs*) in *Brassica napus* under different phosphate (Pi) levels. Differential expression of *BnaNHX1s* (**A**), *BnaNHX2s* (**B**), *BnaNHX4s* (**C**), *BnaNHX6s* (**D**), *BnaNHX7s* (**E**), and *BnaNHX8s* (**F**) under high Pi and low Pi levels. For the transcriptional analysis, the 7 d-old uniform *B. napus* seedlings after seed germination were first hydroponically grown under 250 μM KH_2_PO_4_ (+Pi) for 10 d, and then were transferred to 5 μM KH_2_PO_4_ (-Pi) for 3 d until sampling. The shoots and roots were individually sampled, and each sample includes three independent biological replicates. The significance level of *P* < 0.05 is used as the threshold to identify the differential expression of *BnaNHXs* under high Pi and low Pi conditions. The differentially expressed genes with higher expression are indicated with asterisks.

A previous study has found that nitrate enhances plant SSR more than ammonium does under salt stress [41]. Therefore, we analyzed the transcriptional expression of *BnaNHXs* under nitrate and ammonium conditions to investigate their roles in the plant SSR (Figure 8). Under different forms of nitrogen conditions, we identified a total of seven *BnaNHX* DEGs, which included four *BnaNHX1s*, *BnaA5.NHX2*, *BnaA7.NHX6a*, and *BnaA9.SOS1* (Figure 8). Among these DEGs, we found that only the four *BnaNHX1* DEGs showed higher expression levels under nitrate supply than under ammonium supply (Figure 8). However, the DEGs of *BnaNHX2s, BnaNHX6s* and *BnaSOS1s* were upregulated when ammonium was supplied (Figure 8B,E,F). Another previous report found that plant salt stress damages can be alleviated by phosphorus limitation [42]. Therefore, we analyzed the transcriptional expression of *BnaNHXs* under low phosphate to investigate their roles in the plant SSR (Figure 9). Under phosphate limitation condition, a total of seven *BnaNHX* DEGs, including three *BnaNHX1s* (*BnaA9.NHX1*, *BnaC2.NHX1b*, and *BnaC9.NHX1*), two *BnaNHX2s*, and two *BnaNHX8s*, were identified in the shoots or roots (Figure 9). In detail, the expression of three *BnaNHX1* DEGs was upregulated only in the shoots (Figure 9A), whereas the expression of two *BnaNHX2* DEGs was upregulated in both the shoots and roots (Figure 9B). However, the expression of two *BnaNHX8* DEGs was repressed by low phosphate in the shoots (Figure 9F).

**Figure 10:**
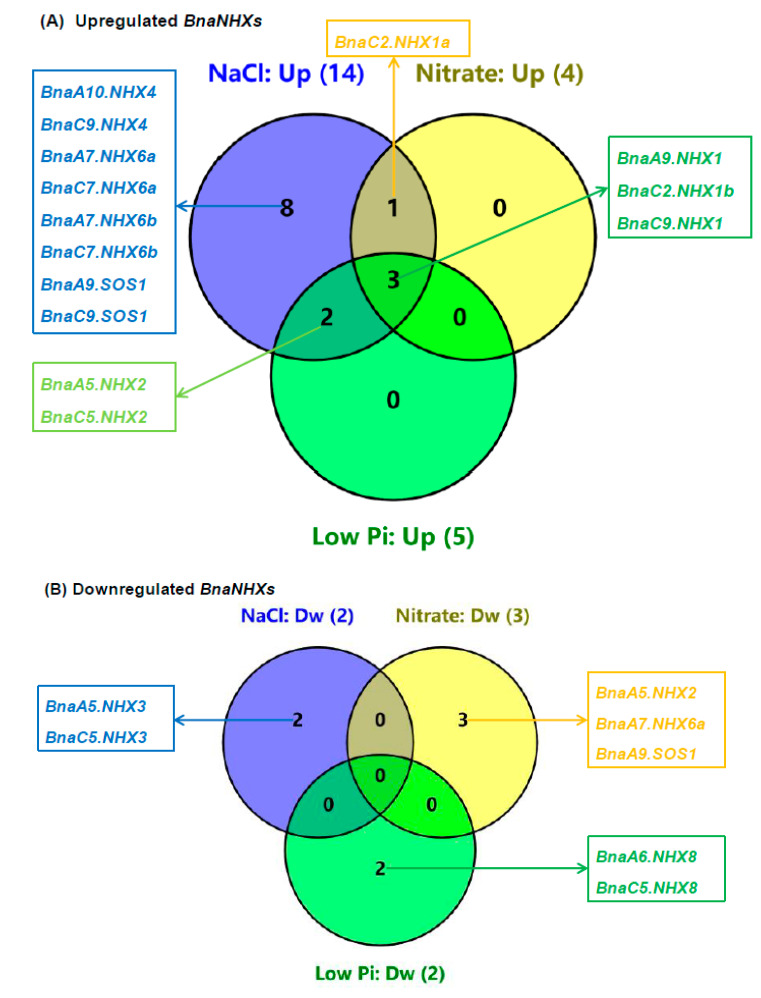
Venn diagrams summarizing the transcriptional responses of the *Na+/H+ antiporters* (*NHXs*) in *Brassica napus* under diverse nutrient stresses. (**A**,**B**) Upregulated (**A**) and downregulated (**B**) *BnaNHXs* in the rapeseed plants under different nutrient conditions. The differentially expressed genes between different nutrient treatments were listed in the brackets. Up, upregulation; Dw, downregulation.

Based on the transcriptional results above, we constructed Venn diagrams to summarize the transcriptional responses of *BnaNHXs* to salt stress, nitrate supply, and low phosphate (Figure 10). The result showed that three *BnaNHX1s* (*BnaA9.NHX1*, *BnaC2.NHX1b*, and *BnaC9.NHX1*) were consistently upregulated under the above three nutrient conditions (Figure 10A). A *BnaNHX* gene (*BnaC2.NHX1a*) was upregulated under both salt stress and nitrate supply conditions, and two *BnaNHX2s* (*BnaA5.NHX2* and *BnaC5.NHX2*) were upregulated under both salt stress and low phosphate conditions (Figure 10A). By contrast, none of the *BnaNHXs* were consistently downregulated under salt stress, nitrate supply, and low phosphate conditions (Figure 10B).

## 3. Discussion

Previous studies have shown that *NHXs* play critical roles in the regulation of plant growth, development, and abiotic and biotic stress responses. Under salt stresses, *NHXs* play pivotal roles in the intracellular Na^+^ excretion and vacuolar Na^+^ compartmentalization, which are important for plant SSR [6,10,17,43]. However, few systematic studies on *NHXs* have been performed in *B. napus* before. In this study, we identified a total of 18 full-length *NHXs*, representing seven subgroups (*NHX1-NHX8* without *NHX5*) in the rapeseed genome. Subsequently, we performed analyses of their phylogeny relationships, physio-chemical characteristics, gene/protein structures, conserved motifs, selection pressure, promoter enriched cis-elements, and differential expression of *BnaNHX*s under different nutrient conditions.

### 3.1. An Integrated Bioinformatics Analysis Provided Comprehensive Insights into the Molecular Features of BnaNHXs

Through the identification of genome-wide *NHX*s in *A. thaliana* and *Brassica* crops, we found that *NHX5* has been lost in *B. rapa*, *B. oleracea*, and *B. napus* (Figure 1B). We proposed that the loss of *NHX5* in the evolutionary process may be attributed to the redundant functions between *NHX5* and *NHX6*, the latter having four homologs in allotetraploid rapeseed (Figure 1B). Bioinformatics analysis showed that *BnaNHXs* can be divided into three classes according to their vacuolar (*BnaNHX1s-4s*), endosomal (*BnaNHX6s*), and plasma membrane (*BnaSOS1s* and *BnaNHX8s*) localizations. The structural diversity of exons/introns provides some evidence for phylogenetic grouping [24]. In *B. napus*, *BnaNHX1s-4s* have fewer exons (13–14) than *BnaNHX6* (21–24), *BnaSOS1* (23), and *BnaNHX8* (14–19) class members (Supplementary Figure S6). Similarly, in soybean, seven members of *GmNHXs* contained 14–15 exons, whereas three other members had 20 exons [44]. These results indicated that there is structural diversity in the *NHX* family in different plant species.

Besides, the conserved motif analysis showed that all the BnaNHXs shared multiple motifs, and BnaNHX1s-4s had amiloride-binding sites (FFIYLLPPI) and transmembrane pores (Figure 4), indicating that BnaNHXs were relatively conserved in the evolutionary process. The SOS pathways, consisting of SOS3, SOS2, and SOS1/NHX7, have been well defined as crucial pathways to control cellular ion homeostasis, by extruding Na^+^ to the extracellular space, thus conferring SSR in plants [43]. Protein interaction network analysis showed that most NHXs interacted with SOS3, SOS2, and some cation/H^+^ antiporters (CHXs), such as the high-affinity K^+^ transporters (HKTs) (Figure 5). This result suggested that the SOS3-SOS2-CHX pathway might be not only a common but also an essential pathway regulating plant SRR. In addition, the cis-acting regulatory elements act as the agents involved in gene activity to control biological processes, such as hormonal responses and abiotic stress responses [35,36]. In the promoter regions of *BnaNHXs*, there were numerous binding sites of transcription factors (Figure 6), such as WRKYs, that respond to plant defense response and participate in plant hormone signaling pathways.

### 3.2. Transcriptional Analysis Revealed Differential Responses and Core Regulators of BnaNHXs Under Salt Stress

In this study, we found that most *BnaNHXs* were significantly upregulated under salt stress, among which the upregulated levels of *BnaNHX1s* were the highest (Figure 7). This finding highlighted the crucial role of *BnaNHX1s* in the regulation of rapeseed SSR. It has been demonstrated that *NHXs* play key roles in chelating Na^+^ into vacuoles to maintain Na^+^ homeostasis, and thus improving plant SSR [45]. In addition, we also found that the expression of *BnaNHXs* in the shoots was significantly higher than that in the roots under salt stress, which might be attributed to the excessive accumulation of Na^+^ in the shoots under high salt concentrations [46]. Based on the differential expression and co-expression network analysis under salt stress (Figure 7A), we proposed that *BnaC2.NHX1a* was the central gene in the vacuolar sequestration of excess Na^+^. In this study, salt stress significantly increased the expression of *BnaSOS1s/BnaNHX7s* (Figure 7G), whereas the plasma membrane-localized *BnaNHX8s* were not responsive to salt stress (Figure 7H). This result suggested that *BnaSOS1s/BnaNHX7s* played more important roles in expelling Na^+^ from intracellular parts than *BnaNHX8s* did.

### 3.3. BnaNHX1s and BnaNHX2s Might be Involved in the Nitrate- and Low Phosphate-Mediated Salt Stress Resistance Enhancement

A previous study has found that nitrate enhances plant SSR more than ammonium does, and the nitrate-mediated plant SSR was thought to be associated with apoplast Na^+^ concentrations [41]. Besides, plant salt stress damages can be alleviated by phosphate limitation, which was thought to increase tissue mass density, enhance osmolytes accumulation, and inhibit Na^+^ uptake [42]. However, whether *NHXs* were involved in the nitrate- and low phosphate-mediated SSR enhancement has been unknown. Therefore, in this study, we investigated the *BnaNHX* expression under different nitrogen forms and phosphate limitation (Figure 8 and Figure 9). It should be acknowledged that the upregulation of *BnaNHXs*, no matter where they were localized, was favorable for plant SSR through increasing Na^+^ compartmentation or efflux. Under different nitrogen forms, among the seven *BnaNHX* DEGs, only *BnaNHX1s* were upregulated, whereas other DEGs, including *BnaA5.NHX2*, *BnaA7.NHX6a*, and *BnaA9.SOS1*, were downregulated (Figure 8). Thus, we proposed that it was *BnaNHX1s*, instead of other *BnaNHXs*, that might be involved in the nitrate-mediated SSR enhancement. There were only three *BnaNHX1s*, two *BnaNHX2s*, and two *BnaNHX8s* responsive to low phosphate, among them, the expression of *BnaNHX1s* and *BnaNHX2s* was induced whereas the expression of *BnaNHX8s* was repressed (Figure 9). Thus, we proposed that both *BnaNHX1s* and *BnaNHX2s* might mediate low phosphate-induced SSR enhancement. Taken together, we proposed that nitrate or low phosphate enhanced plant SSR mainly through increasing vacuolar Na^+^ sequestration.

In conclusion, we believe that global landscapes of *BnaNHXs* will give an integrated understanding of their family evolution and molecular characteristics, which will provide elite gene resources for the genetic improvement of plant SSR through regulating the *NHX*-mediated Na^+^ transport.

## 4. Materials and methods

### 4.1. Retrieval of NHX Sequences

Using the amino acid sequences of Arabidopsis *NHXs* as source sequences, we conducted BLASTp analyses to search for the *NHX* homologs in *B. rapa*, *B. oleracea*, and *B. napus*. In this study, we retrieved the *NHX* sequences using the following databases: The Arabidopsis Information Resource (TAIR10, https://www.arabidopsis.org/) for *A. thaliana*, the *Brassica* Database (BRAD) v. 1.1 (http://brassicadb.org/brad/) [47] for *B. rapa*, Bol base v. 1.0 (http://119.97.203.210/bolbase/index.html) for *B. oleracea* [48], *Brassica napus* pan-genome information resource (BnIR) and Genoscope (http://www.genoscope.cns.fr/brassicanapus/) for *B. napus* [29], National Center for Biotechnology Information (NCBI, www.ncbi.nlm.nih.gov), *EnsemblPlants* (http://plants.ensembl.org/index.html), and Phytozome v. 10 (http://phytozome.jgi.doe.gov/pz/portal.html) [49].

### 4.2. Gene Nomenclature of NHXs in B. napus

In this study, based on the nomenclature previously proposed [50,51,52], we named *NHXs* in *Brassica* species following the criterion: genus (one capital letter) + plant species (two lowercase letters) + chromosome (followed by a period) + name of the *NHX* homologs in *A. thaliana*. For example, *BnaA1.NHX1* represents an Arabidopsis *NHX1* homolog on the chromosome A1 of *B. napus*.

### 4.3. Physical Mapping and Family Expansion Analysis of BnaNHXs

We determined the genomic locations of *BnaNHXs* by BLASTn searches using the complete nucleotide sequences of *AtNHXs*. Using the genomic annotation, we physically mapped the *AtNHXs* and *BnaNHXs* onto the chromosomes using the MapGene2Chromosome v2.1 (http://mg2c.iask.in/mg2c_v2.1/). In this study, we defined the tandem duplicated genes as an array of two or more *NHXs* within a 100-kb genomic region.

### 4.4. Sequence Alignment and Phylogeny Analysis of BnaNHXs

We aligned the full-length protein sequences of the NHXs of Arabidopsis and *B. napus* using ClustalW [53] within MEGA (Molecular Evolutionary Genetics Analysis) v. 6.06 (http://www.megasoftware.net/) [54]. After these alignments, we constructed phylogenetic trees with the neighbor-joining (NJ) method [55]. We set the Poisson correction, pairwise deletion, and bootstrapping (1000 replicates; random seeds) as the required parameters.

### 4.5. Analysis of Evolutionary Selection Pressure and Functional Divergence of BnaNHXs

To analyze positive or negative (purifying) selection pressure on *BnaNHX*s, we calculated the values of synonymous (Ks) and non-synonymous (Ka) nucleotide substitution, and Ka/Ks. First, we performed pairwise alignment of the *BnaNHX* coding sequences (CDSs) using Clustal Omega (http://www.clustal.org/omega/) [56]. Then, we submitted the alignment results to the Ka/Ks Calculator (http://www.bork.embl.de/pal2nal/) software [57] for the calculation of the Ka, Ks, and Ka/Ks with the yn00 method [58]. According to Darwin’s theory of evolution, we usually propose that Ka/Ks > 1.0 means positive selection, while Ka/Ks < 1.0 indicates the occurrence of purifying selection, and Ka/Ks = 1.0 denotes neutral selection. Further, we estimated the divergence time of *BnaNHXs* from their progenitors applying the following formula: T = Ks/2λ, λ = 1.5 × 10^-8^ for Brassicaceae species [59].

### 4.6. Molecular Characterization of BnaNHXs

We used the ExPASy ProtoParam (http://www.expasy.org/tools/protparam.html) [60] program to calculate amino acid number and composition, molecular weight (MW, kD), theoretical isoelectric point (pI), grand average of hydropathy (GRAVY), and instability index (II) of BnaNHXs. Values of II >40.0 suggest that the proteins are unstable [61].

We used the online WoLF PSORT (http://www.genscript.com/wolf-psort.html) [62] program to predict the subcellular localization of BnaNHXs. To identify the transmembrane helices of the AtNHXs and BnaNHXs, we submitted their amino acid sequences to the TMHMM v. 2.0 (http://www.cbs.dtu.dk/services/TMHMM/) program. Phosphorylation sites of BnaNHXs were predicted by NetPhos 3.1 server (http://www.cbs.dtu.dk/services/NetPhos/) [63].

We employed the online SignalP v. 4.1 (http://www.cbs.dtu.dk/services/SignalP/) [64] to predict the presence and location of signal peptide cleavage sites in the BnaNHX protein sequences. To determine the recombinant protein solubility, we used the Recombinant Protein Solubility Prediction (RPSP) v. 2009 (http://biotech.ou.edu) program, which assumes that the NHX proteins are overexpressed in *Escherichia coli* [65].

We used the STRING (Search Tool for Recurring Instances of Neighboring Genes) v 11.0 (https://string-db.org) [66] web-server to retrieve and display the repeatedly occurring association networks of the NHXs. The three-dimensional (3-D) structure of BnaNHXs was predicted using phyre2 (http://www.sbg.bio.ic.ac.uk/phyre2/webscripts/jobmonitor) [67].

### 4.7. Conserved Motif Identification of BnaNHXs

To further examine the structural divergence among the NHXs in *A. thaliana* and *Brassica* crops, we submitted their protein sequences to the online MEME (Multiple Expectation maximization for Motif Elicitation) v. 4.12.0 (http://meme-suite.org/tools/meme) [68] for the characterization of conserved motifs/domains. We used all the default parameters except for the following: the optimum motif width was set to 6–50 bp and the maximum number of motifs was set as 15. The conserved motif sequences were presented by the online Weblogo (https://weblogo.berkeley.edu/logo.cgi) [69].

### 4.8. Elucidation of Gene Structure and Promoter Regulatory Elements of BnaNHXs

Full-length genomic DNA (gDNA) and CDS sequences were collected from the annotated genomes of *A. thaliana* and *B. napus*, and they were used to predict the exon–intron structures of *NHXs*. For each *NHX*, a 2.0-kb genomic sequence upstream from the start codon (ATG) was downloaded from the TAIR (https://www.arabidopsis.org/) website and *B. napus* Genome Browser (http://www.genoscope.cns.fr/brassicanapus/) [29]. Subsequently, we submitted these sequences to the PLACE v. 30.0 (http://www.dna.affrc.go.jp/PLACE/) program [70] to identify putative CREs.

### 4.9. Transcriptional Characterization of BnaNHXs Under Diverse Nutrient Conditions

In this study, the gene expression patterns of *AtNHXs* were obtained from the TAIR eFP Browser [71]. To further investigate the transcriptional responses of *BnaNHXs* under diverse nutrient stresses, we transplanted the uniform *B. napus* seedlings (cv. Zhongshuang 11) into black plastic containers holding 10 L Hoagland nutrient solution. The basic nutrition solution contained 1.0 mM KH_2_PO_4_, 5.0 mM KNO_3_, 5.0 mM Ca(NO_3_)_2_·4H_2_O, 2.0 mM MgSO_4_·7H_2_O, 0.050 mM EDTA-Fe, 9.0 µM MnCl_2_·4H_2_O, 0.80 µM ZnSO_4_·7H_2_O, 0.30 µM CuSO_4_·5H_2_O, 0.37 µM Na_2_MoO_4_·2H_2_O, and 46 µM H_3_BO_3_. The rapeseed seedlings were cultivated in an illuminated chamber with the following growth conditions: the light intensity of 200 μmol m^-2^ s^-1^, the temperature of 25 °C daytime/22 °C night, the light period of 16 h photoperiod/8 h dark, the relative humidity of 70%.

For the salt stress treatment, the 7 d-old uniform *B. napus* seedlings after seed germination were hydroponically cultivated in a NaCl-free nutrient solution for 10 d, and then were transferred to 200 mM NaCl for 1 d until sampling. For the nitrate and ammonium treatment, the 7 d-old uniform *B. napus* seedlings after seed germination were first hydroponically cultivated under 6.0 mM nitrate (NO_3_^-^) for 10 d, and then were transferred to an N-free nutrient solution for 3 d. Subsequently, the above seedlings were sampled after exposure to 6.0 mM ammonium (NH_4_^+^) for 3 d. For the inorganic phosphate (Pi) starvation treatment, the 7 d-old uniform *B. napus* seedlings after seed germination were first hydroponically grown under 250 μM KH_2_PO_4_ for 10 d, and then were transferred to 5 μM KH_2_PO_4_ for 3 d. The shoots and roots were individually harvested and immediately stored at –80 °C until RNA isolation. Each sample contained 3 independent biological replicates for the transcriptional analyses of *BnaNHXs* under diverse nutrient conditions.

### 4.10. Quantitative Reverse-Transcription PCR Assays

The quantitative reverse-transcription polymerase chain reaction (qRT-PCR) assays were used to investigate the relative expression of *BnaNHXs*. After removing genomic DNA with RNase-free DNase I in the RNA samples, total RNA was used as the template for cDNA synthesis with the PrimeScript^TM^ RT reagent Kit with gDNA Eraser (Perfect Real Time) (TaKaRa, Shiga, Japan). We quantified the *BnaNHX* expression using the SYBR^®^ *Premix Ex Taq*^TM^ II (Tli RNaseH Plus) (TaKaRa, Shiga, Japan) kit under an Applied Biosystems StepOne^TM^ Plus Real-time PCR System (Thermo Fisher Scientific, Waltham, MA, USA). The thermal recycle regimes were as follows: 95 °C for 3 min, followed by 40 cycles of 95 °C for 10 s, then 60 °C for 30 s [72]. We also conducted a melt curve analysis to ensure the primer (Table S1) gene-specificity: 95 °C for 15 s, 60 °C for 1 min, 60–95 °C for 15 s (+0.3 °C per cycle). We used the public genes *BnaEF1-α* [73] and *BnaGDI1* [74] as internal references and calculated the *BnaNHX* expression levels with the 2^-ΔΔC^*_T_* method [75]. *p* < 0.05 was used as the significance level to identify the differential gene expression between the treatments and controls.

## Abbreviation

AtArabidopsis thalianaBnaBrasssica napusBolBrassica oleraceaBraBrassica rapaBRAD*Brassica* DatabaseCDSCoding sequenceCRE*cis*-acting regulatory elementDEGsdifferentially expressed genesgDNAgenomic DNAMEMEMultiple expectation maximization for motif elicitationMWMolecular weightNa^+^sodium ionNnitrogenNCBINational Center for Biotechnology InformationNH_4_^+^ammoniumNHXNa^+^/H^+^ antiporterNO_3_^-^nitratePMPlasma membranePPIProtein-protein interactionPiphosphateqRT-PCRquantitative reverse-transcription polymerase chain reactionSOSSalt overly sensitiveSSRsalt stress resistanceTAIRThe Arabidopsis Information ResourceTFtranscription factorTMTransmembrane

## Figures and Tables

**Table 1:** Copy number of the *Na^+^/H^+^ antiporter* (*NHX*) genes in Arabidopsis and *Brassica* crops.

Gene Name	*Arabidopsis thaliana* (125 Mb)	*Brassica rapa* (465 Mb)	*Brassica oleracea* (485 Mb)	*Brassica napus* (1130 Mb)
*NHX1*	1	2	2	4
*NHX2*	1	1	1	2
*NHX3*	1	1	1	2
*NHX4*	1	1	1	2
*NHX5*	1	0	0	0
*NHX6*	1	1	1	4
*SOS1/NHX7*	1	1	1	2
*NHX8*	1	1	1	2
Total	8	8	8	18

**Table 2:** Molecular characterization of the Na^+^/H^+^ antiporters (NHXs) in *Arabidopsis thaliana* and *Brassica napus.*

Gene ID	Gene Name	Block	CDS (bp)	Exon/ Intron	Amino Acid (aa)	Ka	Ks	Ka/Ks	Divergent Time (Mya)
At5g27150	*AtNHX1*	Q	1617	13/12	538				
BnaA09g03600D	*BnaA9.NHX1*	Q	1596	13/12	531	0.0357	0.4543	0.0787	13.10
BnaC02g39630D	*BnaC2.NHX1a*	Q	1635	13/12	544	0.0338	0.3803	0.089	13.29
BnaC02g39600D	*BnaC2.NHX1b*	Q	1635	13/12	544	0.0366	0.4048	0.0903	13.48
BnaC09g02990D	*BnaC9.NHX1*	Q	1608	13/12	535	0.0423	0.4996	0.0848	12.49
At3g05030	*AtNHX2*	F	1641	14/13	546				
BnaA05g32130D	*BnaA5.NHX2*	F	1641	14/13	546	0.0381	0.4029	0.0946	15.69
BnaC05g46790D	*BnaC5.NHX2*	F	1650	14/13	549	0.0357	0.4697	0.076	16.76
At5g55470	*AtNHX3*	W	1590	14/13	529				
BnaA10g09580D	*BnaA10.NHX3*	W	1590	14/13	529	0.044	0.3738	0.1178	15.59
BnaC09g31970D	*BnaC9.NHX3*	W	1602	14/13	533	0.0482	0.3807	0.1265	15.96
At3g06370	*AtNHX4*	F	1512	13/12	503				
BnaA05g30980D	*BnaA5.NHX4*	F	1677	13/12	558	0.0751	0.4801	0.1564	13.15
BnaC05g45460D	*BnaC5.NHX4*	F	1683	13/12	560	0.0728	0.476	0.1529	12.88
At1g54370	*AtNHX5*	C	1566	21/20	521				
At1g79610	*AtNHX6*	E	1608	22/21	535				
BnaA07g20250D	*BnaA7.NHX6a*	E	1674	21/20	557	0.0449	0.3825	0.1174	18.26
BnaA07g35030D	*BnaA7. NHX6b*	E	1935	22/21	644	0.037	0.3676	0.1007	15.22
BnaC06g19720D	*BnaC6. NHX6a*	E	1674	21/20	557	0.0439	0.384	0.1144	17.99
BnaC06g39970D	*BnaC6. NHX6b*	E	1752	24/23	583	0.0472	0.3961	0.1191	14.56
At2g01980	*AtSOS1*	K	3441	23/22	1146				
BnaA09g18880D	*BnaA9.SOS1*	K	3420	23/22	1139	0.0864	0.3656	0.2364	16.55
BnaC09g52290D	*BnaC9.SOS1*	K	3420	23/22	1139	0.0909	0.3756	0.242	18.16
At1g14660	*AtNHX8*	A	2271	20/19	756				
BnaA06g09480D	*BnaA6.NHX8*	A	2232	19/18	743	0.0837	0.2542	0.3291	11.81
BnaC05g10850D	*BnaC5.NHX8*	A	1599	14/13	532	0.0801	0.2814	0.2847	17.52

Note: CDS, coding sequence; Ka, non-synonymous nucleotide substitution rate; Ks, synonymous nucleotide substitution rate.

**Table 3:** Molecular characterization of the Na^+^/H^+^ antiporters (NHXs) in *Arabidopsis thaliana* and *Brassica napus.*

Gene ID	Gene Name	pI	MW (kDa)	II	GRAVY	TM Domains		Subcellular Localization
At5g27150	*AtNHX1*	6.73	59.5	32.71	0.458	12		Vac
BnaA09g03600D	*BnaA9.NHX1*	7.76	58.9	35.14	0.4543	12		Vac
BnaC02g39630D	*BnaC2.NHX1a*	7.50	60.3	34.95	0.3803	12		Vac
BnaC02g39600D	*BnaC2.NHX1b*	7.50	60.4	34.89	0.4048	12		Vac
BnaC09g02990D	*BnaC9.NHX1*	7.50	59.3	34.44	0.4996	12		Vac
At3g05030	*AtNHX2*	8.14	60.5	36.74	0.465	12		Vac
BnaA05g32130D	*BnaA5.NHX2*	7.47	60.6	38.24	0.4029	12		Vac
BnaC05g46790D	*BnaC5.NHX2*	7.49	61.0	37.62	0.4697	11		Vac
At5g55470	*AtNHX3*	8.17	58.9	32.12	0.485	11		Vac
BnaA10g09580D	*BnaA10.NHX3*	7.09	59.0	34.00	0.3738	11		Vac
BnaC09g31970D	*BnaC9.NHX3*	7.07	58.7	34.11	0.3807	11		Vac
At3g06370	*AtNHX4*	6.92	55.6	39.49	0.594	10		Vac
BnaA05g30980D	*BnaA5.NHX4*	7.07	61.8	38.63	0.4801	12		Vac
BnaC05g45460D	*BnaC5.NHX4*	7.07	62.2	39.76	0.476	12		Vac
At1g54370	*AtNHX5*	4.98	57.3	42.99	0.444	10		Endo
At1g79610	*AtNHX6*	5.68	59.3	40.30	0.377	9		Endo
BnaA07g20250D	*BnaA7.NHX6a*	5.62	62.3	41.65	0.3825	10		Endo
BnaA07g35030D	*BnaA7.NHX6b*	6.68	72.4	39.36	0.3676	12		Endo
BnaC06g19720D	*BnaC6.NHX6a*	5.83	62.4	42.95	0.384	10		Endo
BnaC06g39970D	*BnaC6.NHX6b*	6.91	65.5	38.43	0.3961	10		Endo
At2g01980	*AtSOS1*	7.62	127.2	38.60	0.098	9		PM
BnaA09g18880D	*BnaA9.SOS1*	7.02	125.8	34.99	0.3656	12		PM
BnaC09g52290D	*BnaC9.SOS1*	7.00	125.8	35.07	0.3756		12	PM
At1g14660	*AtNHX8*	6.58	83.5	26.66	0.273		12	PM
BnaA06g09480D	*BnaA6.NHX8*	7.53	60.0	24.53	0.2542		8	PM
BnaC05g10850D	*BnaC5.NHX8*	7.34	82.5	26.00	0.2814		9	PM

Note: GRAVY, grand average of hydropathy; II, instability index; MW, molecular weight; pI, isoelectric point; PM, plasma membrane; TM, transmembrane; Vac, vacuole; Endo, endosome.

## Supplementary Materials

Supplementary materials can be found at https://www.mdpi.com/1422-0067/21/10/3429/s1.
