# Supplementary material for: Global Landscapes of the Na+/H+ Antiporter (NHX) Family Members Uncover their Potential Roles in Regulating the Rapeseed Resistance to Salt Stress
Source: Int J Mol Sci. 2020 May 12;21(10):3429. doi: 10.3390/ijms21103429 (PMC7279160; doi:10.3390/ijms21103429)
Supplement: Supplementary file 1 [file ijms-21-03429-s001.zip › ijms-795488-for publish supplementary/Supplementary Figures.docx]

**Supplementary Figures**

**Supplementary Figure S1** Cell-specific expression patterns of the *Na^+^/H^+^ antiporter* (*NHX*) genes in *Arabidopsis thaliana*. (A-H) Relative expression abundances of *AtNHX1* (A), *AtNHX2* (B), *AtNHX3* (C), *AtNHX4* (D), *AtNHX5* (E), *AtNHX6* (F), *AtSOS1/AtNHX7* (G) and *AtNHX8* (H) in cellular components. The red and yellow color indicates relative high and low expression levels of AtNHXs.

**Supplementary Figure S2** Trans-membrane characterization of the *Na^+^/H^+^ antiporter* (*NHX*) proteins in *Arabidopsis thaliana* and *Brassica napus*. The TMHMM (http://www.cbs.dtu.dk/services/TMHMM/) tool was used to predict the transmembrane topology of the AtAAP and BnaNHX proteins.

**Supplementary Figure S3** Phosphorylation sites of the Na^+^/H^+^ antiporter (NHX) proteins in *Arabidopsis thaliana* and *Brassica napus*. The NetPhos (http://www.cbs.dtu.dk/services/NetPhos/) 3.1 server was used to predict the presence and location of phosphorylation sites in amino acid sequences of the AtNHX and BnaNHX proteins.

**Supplementary Figure S4** Characterization of signal peptides of the Na^+^/H^+^ antiporter (NHX) proteins in *Arabidopsis thaliana* and Brassica napus. The SignalP (http://www.cbs.dtu.dk/services/SignalP/) 4.0 server was used to predict the presence and location of signal peptide cleavage sites in amino acid sequences of the AtNHX and BnaNHX proteins.

**Supplementary Figure S5** The secondary structure of the Na^+^/H^+^ antiporters (NHX) proteins in *Arabidopsis thaliana* and *Brassica napus*. The Phyre2 (http: //www.sbg.bio.ic.ac.uk/phyre2/webscripts/jobmonitor) was used to predict the secondary structure in amino acid sequences of the AtNHX and BnaNHX proteins.

**Supplementary Figure S6** Gene structure of the Na^+^/H^+^ antiporter (NHX) genes in *Brassica napus*. Boxes indicate exons, and lines indicate introns.
