# Supplementary material for: Global Landscapes of the Na+/H+ Antiporter (NHX) Family Members Uncover their Potential Roles in Regulating the Rapeseed Resistance to Salt Stress
Source: Int J Mol Sci. 2020 May 12;21(10):3429. doi: 10.3390/ijms21103429 (PMC7279160; doi:10.3390/ijms21103429)
Supplement: Supplementary file 1 [file ijms-21-03429-s001.zip › ijms-795488-for publish supplementary/Supplementary Tables.docx]

**Supplementary Table S1 The gene-specific primer sequences used for qRT-PCR assays in this study**

| Gene name | Forward primer (5´-3´) | Reverse primer (5´-3´) |
| --- | --- | --- |
| *BnaA9.NHX1* | GATCACGCCTCTGTGGTTTCC | CAACAGTTCCAATAGCACCAAAA |
| *BnaC9.NHX1* | TTCTGAACCAAGATGAGACACCTC | GGTCAGTGGAGTGTCTTCCG |
| *BnaC2.NHX1a* | CACTCAATCTCTTTGTTGCACTTCTC | CACCAAAGAGCATAATAGTCACAAAA |
| *BnaC2.NHX1b* | CTCTGTGGTTTCACTCAATCTCTTC | AGATATATGAAGAAAAGATCTTCACTGAAC |
| *BnaA5.NHX2* | GCTTCTCTTTACACCTCCTTAACG | TCACTGAAGACCAATAGATGTGAGC |
| *BnaC5.NHX2* | CATTTCACCTCCTTAACATCGAAG | CCTCTACTAATCAACAAAATAACGACC |
| *BnaA10.NHX3* | ACACTCCTCGGAATTGGTGTG | AGTGATTCTTGAACTCTCTGTCACG |
| *BnaC9.NHX3* | ATTCTGGTGTTTGATGAAGAGCTC | CCCAACTTGGGAAACAGCC |
| *BnaA5.NHX4* | ACCGTATTGGAAAAGTCAGAAACTC | ATGGTCATGAAGTTACGGAAAAAC |
| *BnaC5.NHX4* | GCATTTGGACTACTAAACACCATG | ATATGATCGGTGGAAGAAGATAAATG |
| *BnaA7.NHX6a* | CTTCGCCGTCACAAGTTCTATTAC | GTTTGAAAAGAAAGGTTTGGGC |
| *BnaC6.NHX6a* | ACAACCCAAACCTTTCTTTTCG | CCAAGTTCCTGAAATATAGACAAGACT |
| *BnaA7.NHX6b* | CTTCGCCGTCATAAGTTCTATTATCTA | GATCAAAGGATATAATATGATGGGC |
| *BnaC6.NHX6b* | GATGCTCGTCCTCTCTTTCGTG | TTAGGTTGTAGGCTGAATCCTGAC |
| *BnaA9.SOS1* | GCGACAGGTTCTTCTTCGGAG | TGAGTTCCATACTCGAGAGATCCA |
| *BnaC9.SOS1* | TATAGACTTCTGGAGGAGGCGG | AATCCCGATGACGAGGAGC |
| *BnaA6.NHX8* | GACAGCATTTAAGGGTGAGAACC | ATGGGTACAAAACTCCAACCATTA |
| *BnaC5.NHX8* | TCCTTGATGAATGACGGGGTA | GAATCTAAGCCAAAAAACTGATGT |
